# Prevalence and Determinants of Diabetic Retinopathy Among Type 2 Diabetic Patients in Saudi Arabia: A Systematic Review

**DOI:** 10.7759/cureus.42771

**Published:** 2023-07-31

**Authors:** Eman A Aljehani, Asma E Alhawiti, Rofayda M Mohamad

**Affiliations:** 1 Family Medicine Department, Almuruj Primary Health Care Center, Tabuk, SAU; 2 Preventive Medicine Department, King Fahad Specialist Hospital, Tabuk, SAU; 3 Preventive Medicine Department, King Salman Armed Forces Hospital in Northwestern Region, Tabuk, SAU

**Keywords:** saudi arabia, risk factors, prevalence, type 2 diabetes, retinopathy

## Abstract

Diabetic retinopathy (DR) is a preventable complication of diabetes; however, it is a serious one if not early recognized and properly managed as it can lead to visual impairment. This review aimed to summarize the prevalence and determinants of DR among type 2 diabetic patients in Saudi Arabia. Eligible articles for this systematic review were quantitative observational studies that were English-published between 2015 and 2021, peer-reviewed, and conducted on patients with type 2 diabetes mellitus (DM). The studies were obtained by searching PubMed/Medline, ScienceDirect, Cochrane Library Database, and Google Scholar. The risk of bias was assessed using the National Institutes of Health quality assessment tool for observational cohort and cross-sectional studies. Out of 16 preliminary studies, 12 were eligible for inclusion in this systematic review. One study was a chart review, one was a prospective study, and the remaining were cross-sectional studies. Regarding the study tool, retinopathy was diagnosed by an ophthalmologist using fundus photography and/or slit-lamp examination in most of the studies (n=7). However, some studies reported obtaining data from patient interviews and medical files (n=4). Concerning the outcome, an overall high prevalence of DR (ranging between 6.25% and 88.1%) and some significant associated risk factors were determined, including longer duration of diabetes, older age, poor blood pressure control, poor glycemic control, and physical inactivity. Most studies showed moderate overall quality. In conclusion, DR is a common complication of type 2 diabetes in Saudi Arabia. Some avoidable risk factors are identified, through which the doctors can identify patients at high risk of DR through early screening and can, thus, initiate prompt treatment to reduce the risk of vision deterioration.

## Introduction and background

According to the World Health Organization, diabetes mellitus (DM) is defined as “a metabolic disorder of multiple etiologies characterized by chronic hyperglycemia with disturbances of carbohydrate, fat, and protein metabolism resulting from defects in insulin secretion, insulin action, or both” [1].

Diabetes mellitus is a worldwide chronic health problem affecting more than 366 million persons, and the prevalence will expectedly reach 552 million by the year 2030 [2]. The Kingdom of Saudi Arabia occupies the seventh rank globally regarding the prevalence of diabetes and the second rank in the Middle East. Current statistics indicate that approximately seven million Saudi people are diagnosed with diabetes, and another three million are in the prediabetes stage [3].

Diabetes mellitus causes several complications, including diabetic retinopathy (DR), which affects almost 18.5% of diabetic patients all over the world [4] and is considered a leading cause of blindness in diabetics in the age group 20-74 years [5].

Commonly reported risk factors for DR were older age, higher levels of glycated hemoglobin (HbA1c), dyslipidemia, obesity, longer duration of diabetes, smoking, nephropathy, and high blood pressure [6,7].

In Saudi Arabia, the prevalence of DR ranged from 28.1% to 45.7%, and vision-threatening DR affects 4.5% to 17.5% of patients with diabetes [8]. The global prevalence of DR was estimated to be 22.27% (95% confidence interval (CI), 19.73%-25.03%), according to a meta-analysis that included 59 population-based studies [9]. In the United Kingdom, a study reported a DR prevalence of 38.9% in 2012 which decreased to 36.6% in 2016 [10]. In Denmark, results of the DR screening program showed that the prevalence and five-year incidence rate of DR among type 2 DM patients were 8.8% and 3.8%, respectively [11]. Based on the rates from 41 studies (48,995 patients), a recent meta-analysis [12] found that the prevalence rates of DR, proliferative DR, and non-proliferative DR were, in order, 28%, 6%, and 27% in Asian type 2 DM patients. The meta-analysis found a variation in the prevalence rates of DR among Asian countries, with the highest rates being in India (42%), Malaysia (35%), and Singapore (33%) [12].

Diabetic retinopathy is characterized by signs of retinal ischemia (retinal microvascular abnormalities, intravenous caliber abnormalities, hemorrhages, microaneurysms, neovascularization, and cotton‑wool spots) and/or signs of increased retinal vascular permeability. Depending on signs, retinopathy is grouped into two main categories: non-proliferative diabetic retinopathy (NPDR “mild, moderate, and severe”) and proliferative diabetic retinopathy (PDR). Loss of vision in DR can result from many mechanisms, including neovascularization resulting in vitreous hemorrhage and/or macular edema, retinal detachment, and retinal capillary nonperfusion [13].

The American Diabetes Association recommends screening type 2 diabetic patients for DR at the time of diagnosis of diabetes and then routinely every one or two years if there is no evidence of retinopathy. However, patients showing signs of retinopathy should undergo retinal examinations more frequently, with the time intervals decided according to their stage of DR [14].

Diabetic retinopathy is a preventable complication of diabetes; however, it is a serious one, if not early recognized and properly managed, as it can lead to visual impairment. Unfortunately, most patients are asymptomatic until the very late stages. Consequently, the disease can rapidly progress before the initiation of therapy leading to poor outcomes. Therapy can produce favorable results in the early stages of DR, hence comes the necessity of screening for DR for early identification of at-risk patients who will then benefit from prompt treatment.

There is a need to define the magnitude of the problem of DR in Saudi Arabia to draw the attention of doctors providing care to type 2 diabetic patients so that they become alert for identifying such patients. Also, the risk factors of DM should be defined, both to identify high-risk patients and to change any modifiable risk factors to improve visual outcomes in diabetic patients. Moreover, Saudi health authorities can benefit from the evidence provided by primary and secondary research to assess the feasibility of initiating screening programs for DR among high-risk patients and training doctors in diabetic clinics. Therefore, this systematic review aimed to summarize the prevalence and determinants of DR among type 2 diabetic patients in Saudi Arabia. The objectives of this systematic review included: (a) estimating the prevalence of DR among type 2 diabetic patients in Saudi Arabia and (b) identifying the significant risk/preventive factors that increase/reduce the risk of developing DR among type 2 diabetic patients in Saudi Arabia. 

## Review

Methods

A thorough search was conducted using relevant keywords such as (“Diabetic retinopathy” OR “diabetic complications”) AND (“prevalence” OR “rate” OR “risk factors”) AND “Saudi Arabia” AND (”type 2 diabetes”). Studies were extracted from the online databases of PubMed/Medline, ScienceDirect, Cochrane Library Database, and Google Scholar.

Articles were first screened by abstract, and then the screening of the full-text articles was based on eligibility. Criteria for article selection included: peer-reviewed publications, studies conducted on Saudi patients with type 2 DM, articles that investigated the prevalence and associated factors of DR, studies carried out recently between 2015 and 2021, full articles should be in English, and quantitative observational studies. Other studies were excluded from this systematic review, including ongoing studies, reviews, conference proceedings, and abstracts without full-text studies.

According to the specified inclusion and exclusion criteria, articles were screened, and the data were then extracted and included in this review. The preliminary search yielded 16 articles. Four articles were excluded (one review article, one article on type 1 diabetic patients, and two abstracts without full-text studies).

Primary outcome measures were the prevalence of DR and its risk factors (demographic and diabetes/clinical-related factors).

The risk of bias (ROB) of the included studies was assessed using the National Institutes of Health quality assessment tool for observational cohort and cross-sectional studies [15].

Results

**Figure 1:**
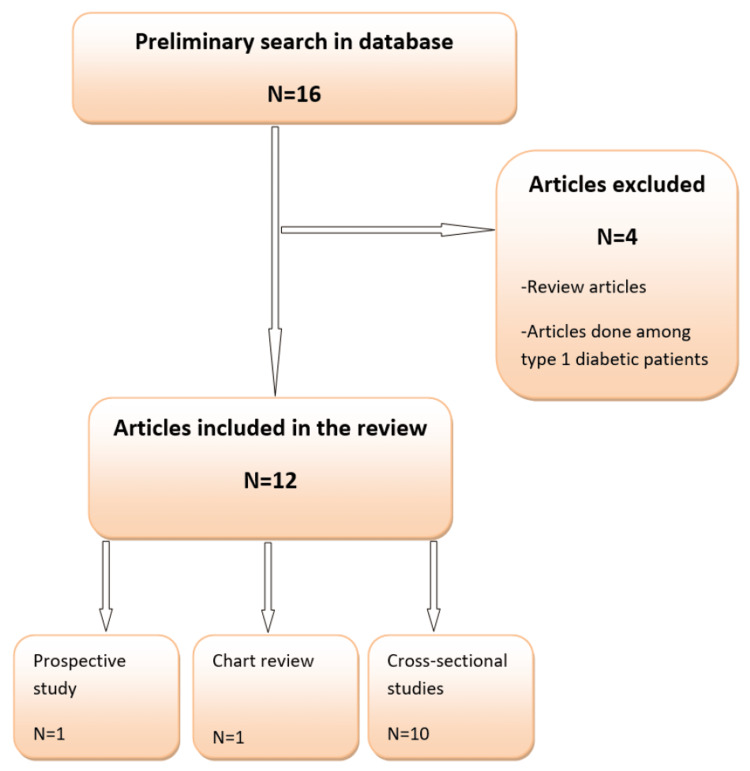
Flow diagram of the studies included in the systematic review. N: number. Note: This image is the author's own creation.

Out of 16 preliminary studies, 12 were found eligible for inclusion in this systematic review. Four articles were excluded due to different reasons, including review articles or including only type-1 diabetic patients (Figure 1).

Only one study was a chart review, and one was a prospective study, whereas the remaining were observational cross-sectional studies. Regarding the study tool, retinopathy was diagnosed by an ophthalmologist using fundus photography and/or slit-lamp examination in most of the studies (n=7). However, some studies reported obtaining data from patient interviews and medical files (n=4), and in one study, the nature of the study tool was not clear. Concerning the outcome, the prevalence of DR and its associated factors, either patients' demographics, diabetes-related factors, or general health-related factors, were assessed in these studies. The main findings revealed an overall high prevalence of DR (ranging between 6.25% and 88.1%), and some significant associated risk factors were determined (Table 1).

**Table 1:** Prevalence and determinants of diabetic retinopathy among diabetic patients in Saudi Arabia (2015-2021). BMI: body mass index; DR: diabetic retinopathy; STDR: sight-threatening diabetic retinopathy, DM: diabetes mellitus; NP: non-proliferative.

Author(s)	Aim(s)	Study design, setting, and sample	Study tool (diagnosis of DR)	Main results	Strengths and limitations
Alrashedi et al. [16]	To estimate the prevalence and identify associated risk factors of DR in type 2 diabetic patients, based on the level of HbA1c.	A cross-sectional study was done among 428 type 2 diabetic patients between January 2019 and February 2020. Patients were divided based on HbA1c into a ‘high-risk group’ (≥9%) and a ‘low-risk group’ (≤7%).	An ophthalmologist assessed the eyes using fundus photography.	The prevalence of DR was 88.1% and 22.1% in high- and low-risk groups, respectively. Macular edema was significantly more prevalent in the high-risk group than in the low-risk group (15.8% vs. 4.9%, p<0.001). High-risk patients had significantly higher HbA1c and low-density lipoprotein levels (p<0.0001) on bivariate analysis but not on multivariate analysis.	The study demonstrated specifically the role of the HbA1c level in the development of DR. However, it has some important limitations. Being a single health facility center, generalizability should be considered with care. In addition, a lot of missing data were observed from medical records, particularly concerning smoking status, BMI, ACE inhibitors, and ARBs use.
Bardisi [17]	To estimate the prevalence and identify risk factors of different diabetic complications, including DR among type 2 diabetic patients attending Primary Health Care Centers in Riyadh.	A cross-sectional study was done among males with T2DM. Data were collected through face-to-face interviews and also from patient medical records.	Data were obtained from patients' interviews and medical files	The study included 351 patients. The prevalence of overall diabetic complications was 65.8%, and that of DR was 30.2%. Significant predictors for diabetic complications were older age (>60 years), smoking, physical inactivity, high HbA1c level, uncontrolled blood pressure, and longer duration of DM.	The study collected data from patients in Riyadh. However, it is not specific for evaluating DR as it evaluated all complications together. Also, the cross-sectional design adopted in this study is considered a limitation. BMI and HbA1c data were collected from the records. Data were collected from only primary healthcare centers in Riyadh City. Only male patients were included in the study. The sample was selected by a non-probability technique.
Alghamdi et al. [18]	To estimate the prevalence and specify the determinants of DR in type 2 diabetes mellitus patients attending the diabetic center.	A cross-sectional study was conducted among 251 type-2 diabetic patients registered at the diabetic center at Al-Noor Specialist Hospital, Makkah City.	Slit-lamp examination and colored fundus photographs were used for the diagnosis and grading of DR.	The prevalence of DR was 54.6%. DR was mild non-proliferative (NP) in 52.6% of patients, severe NP in 15.3%, and proliferative in 4.4%. Predictors for DR were having diabetes for 11 to 16 years, not taking daily medications on time, not going for fundus examination annually and uncontrolled diabetes. In addition, patients not administering insulin had a lower probability by 70% to suffer DR. An increase of one unit in BMI increased the probability of developing DR by 11%.	The study adds to the literature regarding the prevalence and correlation of DR in Saudi Arabia. However, it is a single-center study, which could affect the generalizability of the results.
Al-Esawi and Amer [19]	To assess the prevalence and identify risk factors of diabetic retinopathy among diabetic patients	A cross-sectional study was conducted among diabetic patients attending the ophthalmic clinic in a general hospital in the Madinah region.	Not mentioned	The prevalence of DR was 52.3%. The most common stage was mild (83.9%). The significant risk factors included age, type of diabetes, and co-morbidities.	The study adds to the literature regarding the prevalence and determinants of diabetic retinopathy in Saudi Arabia. Regarding limitations, a relatively small sample size (n=107), being a single-center study, and the method of DR diagnosis was not well described.
Bajaber and Alshareef [20]	To explore the role of lifestyle factors in putting type 2 diabetic patients at a greater risk of macro- and microvascular complications, including DR.	A cross-sectional survey was performed among adults with type 2 diabetes in three major cities in the Kingdom of Saudi Arabia. Data were collected through both patient interviews and review of medical records.	Data were obtained from patient interviews and medical files.	A total of 1121 participants were included. The prevalence of DR was 42.8%. Risk factors associated with DR were age, duration of DM, hypertension, insulin administration, poor glycemic control, inadequate physical activity, and longer sitting time.	The study included a big sample size from different regions. However, it is not specific for evaluating DR as it evaluated all complications together. Also, the cross-sectional design adopted in this study is considered a limitation. The authors depended on patients self-reporting of retina problems for those without ophthalmology examination reports in their files.
Alramadan et al. [21]	To assess the prevalence and define the determinants of DR Saudi population aged over 40 years	A population-based cross-sectional study was conducted between 2014 and 2017 including 890 Saudi diabetics aged >40 years in Riyadh city.	Digital fundus photography was done. Diabetic retinopathy was graded into no DR, NPDR, and PDR. Sight-threatening DR (STDR) included PDR and/or diabetic macular edema	The rate of DR was 44.7%, adjusted for age and sex. Diabetic retinopathy among males was significantly higher than in females and in 60- and over-year-old patients than in 40- to 60-year-old patients. The crude prevalence of STDR was 12.4%. Bilateral and unilateral severe visual impairment affected 1% and 1.8% among patients with DR. Patients who underwent retinal laser treatment for STDR accounted for 6.1%.	It is a population-based survey for blinding eye diseases, including DR in the central semi-urban areas of Saudi Arabia. However, it is limited to patients aged over 40 years in Riyadh, which could impact the generalizability of findings.
Yasir et al. [22]	To determine the prevalence and grading of DR and its risk factors in patients with diabetes attending primary care centers.	A chart review (2014-2017) study was conducted in three randomly selected primary care centers among 250 patients with diabetes. At the initial visit, the ophthalmological findings were recorded. For three successive yearly screenings, the screening results were assessed to estimate the changes that occurred in the prevalence, incidence, and progression of DR, in addition to the degree of association with the most predictive risk factors.	Ophthalmological findings were recorded at baseline and then yearly for three successive years.	Initially, the prevalence of DR was 15.2%, with a steady increase in the prevalence of DR over three consecutive years. Significant associated risk factors for DR were age, duration of DM, uncontrolled diabetes, hypertension, dyslipidemia, nephropathy, and insulin therapy.	The study is characterized by a long period of screening for DR. However, it has some limitations, including the small sample size and baseline differences among the participants regarding the initial presentations as well as the history and complications of diabetes. Additionally, the study setting (primary care centers) differs from a hospital-based setting.
Magliah et al. [23]	To estimate the prevalence of diabetic retinopathy and determine its risk factors as well as to assess the effect of expanding the screening interval on the rate of detecting diabetic retinopathy.	A cross-sectional study was performed by reviewing the medical records of 167 patients recruited from three primary care centers in Jeddah City between April 2015 and April 2018.	Reviewing of medical and ophthalmologic reports of patients.	The prevalence of DR was 21.6%. Patients with DR were significantly older (61 ± 11 vs. 54 ± 10 years P=0.001). In addition, HBA1C level and uncontrolled diabetes, hypertension, insulin use, dyslipidemia, and nephropathy were significant predictors for DR.	The study represents screening for DR at primary care centers, which is important for early detection. In addition, expanding the screening interval up to two to three years was more yielding in detecting more cases and with no increased risks. However, a relatively small sample size could be a limitation of this study.
Al-Zamil [24]	To estimate the period prevalence of DR and identify the risk factors for it in newly diagnosed type 2 diabetes mellitus patients	A prospective study was conducted where 112 newly diagnosed type-2 diabetes mellitus patients between January 2012 and January 2015 were examined for DR	The fundus was examined by a retina consultant after papillary dilatation, using slit‑lamp indirect ophthalmoscopy.	The prevalence of DR was 6.25%. Non-proliferative DR was present in all patients with DR. Two patients (28.6%) had bilateral clinically significant macular edema. The only factor significantly associated with DR was uncontrolled HbA1C levels.	The study investigated DR in newly diagnosed type 2 diabetic patients. However, limitations included a relatively small sample size that may impact statistically significant values.
Ahmed et al. [25]	To measure the prevalence and grades of retinopathy and identify its risk factors in type 2 diabetic patients.	A cross-sectional study included 401 type-2 diabetic patients in Abha diabetic center.	Fundus photographs and slit lamp examination were used to diagnose and grade retinopathy.	The prevalence of retinopathy was 36.4%; of them, 57.5% were mild, 19.9% were moderate, 11% were severe NP, and 11.6% were PR. The rate of maculopathy was 7.2%. Risk factors of DR included older age, early onset, longer duration of diabetes, poor glycemic control, insulin use, and the presence of hypertension, diabetic neuropathy, or nephropathy.	The study provides a picture of the situation in the Abha region. The limitation of this study is that a predictive inference cannot be extracted from this observational data since a more extensive study is required.
Hajar et al. [26]	To estimate the prevalence and associated factors of blindness and DR in the Jazan Region, Southern Saudi Arabia.	A cross-sectional study was conducted among patients aged over 50 years.	Patient interview and ophthalmic assessment of fundus.	The prevalence of DM was 22.4%, and 27.8% among them had DR. Patients with STDR accounted for 5.7%.	The study focused mainly on determining the magnitude and causes of avoidable blindness and visual impairment, and the magnitude of DR.
Al-Rubeaan et al. [27]	To explore DR prevalence and its risk factors in the type-2 diabetes epidemic area.	A cross-sectional study was carried out among 50,464 Saudi type-2 diabetic patients aged 25 years and more.	Using data from the Saudi National Diabetes Registry.	The prevalence of DR was 19.7%; 9.1% had NPDR, 10.6% had PDR, and 5.7% had macular edema. The most significant associated risk factors were the patient's age and duration of diabetes. Other significant risk factors were male gender, neuropathy, nephropathy, insulin use, poor glycemic control, and hypertension, while smoking, hyperlipidemia, and obesity significantly decreased the risk for DR.	The strength of the study was the population-based nature with a large sample size. However, the study is limited by being a retrospective one depending on information from records; particularly those concerning type of DR. No data regarding the degree of visual impairment and blindness and being a cross-sectional study.

The results of the ROB assessment revealed that two studies had a higher ROB (lower methodological quality) [19,23], while the remaining studies had moderate quality with less ROB (Table 2).

**Table 2:** The risk of bias assessment for the included studies was based on the National Institutes of Health (NIH) Quality Assessment Tool for the observational cohort and cross-sectional studies. Q1: Was the research question or objective in this paper clearly stated?; Q2: Was the study population clearly specified and defined?; Q3: Was the participation rate of eligible persons at least 50%?; Q4: Were all the subjects selected or recruited from the same or similar populations (including the same time period)? Were inclusion and exclusion criteria for being in the study prespecified and applied uniformly to all participants?; Q5: Was a sample size justification, power description, or variance and effect estimates provided?; Q6: For the analyses in this paper, were the exposure(s) of interest measured prior to the outcome(s) being measured?; Q7: Was the timeframe sufficient so that one could reasonably expect to see an association between exposure and outcome if it existed?; Q8: For exposures that can vary in amount or level, did the study examine different levels of the exposure as related to the outcome (e.g., categories of exposure, or exposure measured as a continuous variable)?; Q9: Were the exposure measures (independent variables) clearly defined, valid, reliable, and implemented consistently across all study participants?; Q10: Was the exposure(s) assessed more than once over time?; Q11: Were the outcome measures (dependent variables) clearly defined, valid, reliable, and implemented consistently across all study participants?; Q12: Were the outcome assessors blinded to the exposure status of participants?; Q13: Was the loss to follow-up after baseline 20% or less?; Q14: Were key potential confounding variables measured and adjusted statistically for their impact on the relationship between exposure(s) and outcome(s)?; CD: cannot determine; NA: not applicable.

Study	Q1	Q2	Q3	Q4	Q5	Q6	Q7	Q8	Q9	Q10	Q11	Q12	Q13	Q14
Alrashedi et al. [16]	Yes	Yes	CD	Yes	Yes	No	No	Yes	Yes	CD	Yes	CD	NA	Yes
Bardisi [17]	Yes	Yes	Yes	Yes	Yes	No	No	No	Yes	CD	Yes	CD	NA	No
Alghamdi et al. [18]	Yes	Yes	Yes	Yes	Yes	No	No	Yes	Yes	CD	Yes	CD	NA	Yes
Al-Esawi and Amer [19]	Yes	Yes	CD	Yes	No	No	No	No	No	CD	Yes	CD	NA	No
Bajaber and Alshareef [20]	Yes	Yes	Yes	Yes	Yes	No	No	Yes	Yes	CD	Yes	CD	NA	Yes
Alramadan et al. [21]	Yes	Yes	Yes	Yes	Yes	No	No	No	Yes	CD	Yes	CD	NA	No
Yasir et al. [22]	Yes	Yes	Yes	Yes	No	No	No	Yes	Yes	CD	Yes	CD	NA	Yes
Magliah et al. [23]	Yes	CD	CD	Yes	No	No	No	No	No	CD	Yes	CD	NA	No
Al‑Zamil [24]	Yes	Yes	CD	Yes	No	Yes	No	No	Yes	Yes	Yes	CD	CD	No
Ahmed et al. [25]	Yes	Yes	Yes	Yes	Yes	No	No	Yes	Yes	CD	Yes	CD	NA	Yes
Hajar et al. [26]	Yes	Yes	Yes	Yes	Yes	No	No	No	Yes	CD	Yes	CD	NA	No
Al-Rubeaan et al. [27]	Yes	Yes	Yes	Yes	Yes	No	No	Yes	Yes	CD	Yes	CD	NA	Yes

Discussion

The prevalence of DM has increased dramatically in Saudi Arabia over the last few decades. Consequently, the rate of DM-related complications, including DR, is expected to increase [28]. This systematic review summarizes the results concerning the prevalence and determinants of DR in Saudi Arabia between 2015 and 2021.

The prevalence of DR in Saudi Arabia, according to the included studies [16-23,25-27], ranged from 6.25 [24] to 54.6% [18], with rates reaching as high as 88% within high-risk groups. Variable rates were also reported in studies carried out on both regional and international levels. In the United Arab Emirates, a prevalence of 19% has been reported [29], whereas rates of 12%, 20.5%, 26.6%, and 34.1% were reported from Kuwait [30], Egypt [31], Iran [32], and Jordan [33], respectively. However, higher rates were observed in Oman (42.4%) [34] and Jordan (64%) [35]. On the international level, the global prevalence was 34.6% [36]. However, rates of 12.2%, 21.7%, 27.9%, and 40.3% were reported in Spain [37], India [38], China [39], and the United States [40], respectively.

The variations in the rates of DR varied widely among the studies, which may be attributed to differences in the study designs as most studies were cross-sectional in the current systematic review [16-21,23,25-27], while two studies were longitudinal [22,24]. Also, heterogeneity among studies regarding the patients' sociodemographic and clinical characteristics could have a role in explaining this variation in the prevalence rates, particularly the age of the included patients and the duration of DM. The duration of DM varied among the studies and would impact the development of DR; thus, the rate of DR will be lower if patients with short durations of DM were included, as was noticed in the study by Al-Zamil [24]. Another potential contributing factor for this variation is the utilization of different diagnostic tools for diagnosing DR, either clinically through the assessment with a slit-lamp and colored fundus photography or through data obtained from medical records. In addition, the study setting is an important factor for determining the prevalence of DR, as carrying out a study in a specialized diabetic center usually yields higher rates [18,25] compared to primary care settings [17,22,23] and general hospitals.

The current systematic review summarized the identified risk factors for DR among the Saudi population as stated by the included studies. Identification of potential risk factors is of paramount importance in reducing the rate of DR by helping identify the patients at risk to design frequent screening schedules and rigorous management of modifiable risk factors to hinder disease development or progression.

The most notable risk factor was poor glycemic control in univariate [8,17,18,20,22-25,27] and multivariate analyses [18,20,22,27]. Elevated HbA1C levels are known to correlate with a higher risk of DM-related complications such as nephropathy, neuropathy, and retinopathy [41,42]. Good control of glycemic status can reduce the risk of developing vascular complications and/or reduce their severity if they develop [6,43,44]. The effect of strict glycemic control can last for several years after treatment as shown by the extension to the Diabetes Control and Complications Trial [45].

The older age of patients was significantly associated with a higher probability of DR in most studies in univariate analysis [17-23,25,27] and was confirmed as an independent risk factor on multivariate analysis in two studies [22,27]. However, it was found insignificant in multivariate analysis in the study by Bajaber and Alshareef [20]. The age did not differ significantly in two of the studies [16,24], but one study included only newly diagnosed cases with DM [24] and the other assessed the age within risk groups based on the HbA1C level [16]; thus, the effect of age may not become apparent in these two studies. Longer duration of DM increased the risk of DR in univariate [17,18,20,22,25,27] and multivariate analyses [18,20,22,25,27]. Only one study reported a lack of significant correlation between the duration of DM and DR [16], which may be explained by the assessment of risk factors within subgroups of patients. A study in the United States found that each year following the development of DM increases the risk of DR by 6% [13]. The effect of the older patient’s age and longer duration of DM could be explained by the longstanding hyperglycemia, which induces macro- and micro-vascular effects and the aggravation of hyperglycemia when the pancreatic function further deteriorates with age. This explanation may also apply to the effect of insulin treatment found in the current review, as insulin is usually required for the treatment of advanced cases in which oral hypoglycemics do not produce good glycemic control, because of pancreatic deterioration, which is common with older age and after several years of suffering from DM [46]. However, insulin might increase the progression of DR by increasing the permeability of the microvasculature of the retina [47]. Treatment with insulin was found to significantly increase the risk of DR both on univariate [18,20,22,23,25,27] and multivariate analyses [18,20,22,25,27]. All these findings emphasize the need for early diagnosis and strict management of DM to reduce the risk of developing DR or at least delay its onset and severity. Screening programs for DM and raising the Saudi public's awareness about the importance of regular health check-ups, healthy lifestyle practices, and treatment adherence can play a pivotal role in reducing the incidence of DM-related complications, including retinopathy.

Male sex was a risk factor for developing DR in three studies on univariate analysis [18,21,27], but there was no significant sex difference in six studies [16,19,20,22-24]. The presence of sex differences in the risk of DR is a controversial issue, and there is no clear explanation for the observed male predominance in some studies [48].

Hypertension was a significant risk factor of DR in univariate [17-20,22,23,25] and multivariate analyses [20,22]. This finding could be explained partially by the common coexistence of hypertension and DM. In addition, hypertension may contribute to the retinal pathophysiological changes with the formation of retinal hard exudates and hemorrhages [49]. However, two previous clinical trials did not find a significant effect of tight control of blood pressure on the progression of DR [50,51].

The presence of other complications of DM seemed to increase the risk of developing DR, particularly nephropathy [18,22,23,25,27] or neuropathy [25,27]. Their effect was noted in multivariate analysis in one study only [27]. This association is well documented in previous research and reflects the common pathophysiology of these complications, which involve macro- and micro-vascular involvement [52].

Some factors showed conflicting results, such as smoking, dyslipidemia, and obesity, which significantly reduced the risk in the study by Al-Rubeaan et al. [27] on both univariate and multivariate analyses. The association of obesity and body mass index with the development and/or progression of DR is contradictory in the literature [48]. The protective effect of overweight was hypothesized to reflect the observation that patients with poorer glycemic control are usually underweight, while strict glycemic control is commonly associated with increased body weight [48].

The identified risk factors for developing DR among the Saudi population are in accordance with those reported in other populations. A longer duration of DM and a higher body mass index increased the likelihood of developing DR, as shown in studies conducted in the United Arab Emirates [29] and Iran [32]. Noncompliance with the intake of anti-diabetic medications as well as regular annual eye examinations were shown as risk factors for DR [53]. Furthermore, diabetic patients treated with insulin were more likely to develop DR [53,54]. The association between other complications of diabetes such as neuropathy, nephropathy, and cardiovascular diseases on one side and DR on the other side has been documented by earlier studies [55-57]. The association between uncontrolled diabetes and DR has been documented by other researchers [32,57].

The results of this systematic review should be interpreted cautiously in light of the limitations and potential biases observed in the included studies. Limitations of these studies included a lack of generalizability of findings as being single-facility studies or limited to certain patients’ age [16,18,19,21,22], small sample sizes [19,22-24], being cross-sectional studies that doesn't prove causation [17,18,20,25-27], and depended on obtaining information from medical records rather than ophthalmic examination [16,17,27]. Another important limitation was the lack of sample size calculation in several studies [19,22-24]. In addition, some studies did not state the number from which the sample was chosen and whether any eligible subjects were excluded for some reason [16,19,23,24]. Moreover, several studies did not assess different levels of independent factors (e.g., duration of DM, HbA1C levels) on DR [17,19,21,23,24,26]. Another important source of bias is whether the outcome assessors were blinded to the patients’ history and medical condition, which was not stated in any of the studies. Also, half the studies did not perform a multivariate analysis to adjust for confounding factors and find the independent factors increasing the risk of DR [17,19,21,23,24,26]. Future studies should avoid these limitations to achieve high-quality evidence regarding the prevalence of DR in patients with type 2 DM in Saudi Arabia and the associated independent risk factors.

## Conclusions

Overall, the findings from this systematic review indicate that DR is a common complication of type 2 diabetes in Saudi Arabia with a prevalence rate ranging from 6.25 to 54.6%, which is comparable to the reported worldwide rates, despite the variation across the Saudi studies. Several risk factors were identified, including unavoidable factors such as age, gender, and duration of diabetes, while some others are avoidable, such as poor glycemic control, poorly controlled hypertension, obesity, non-compliance with anti-diabetic medications, and annual eye check-ups. The findings of the current systematic review helped to realize the magnitude of the problem in Saudi Arabia and can guide the health authorities in implementing future preventive and management projects. The preventive projects should initiate screening for DR in all high-risk patients and increase the awareness of primary care physicians, family physicians, and specialists in DM to encourage their patients and refer them for routine screening. Also, the identified preventable risk factors should be addressed to hinder the development and/or progression of DR. However, the quality of the available studies is impaired by several limitations in their design and methodology, necessitating the conduction of future longitudinal Saudi research to determine the potential association between various risk factors and the development of DR. Future studies should preferably be prospective cohorts in design, ensure adequate sample size, and sufficient follow-up. Studies should endeavor to assess the less commonly evaluated preventable risk factors, such as smoking and physical activity. In addition, adjustment for confounding factors is of paramount importance to identify the independent risk factors of DR.
